# Non-Invasive Monitoring of Core Body Temperature for Targeted Temperature Management in Post-Cardiac Arrest Care

**DOI:** 10.3389/fmed.2022.810825

**Published:** 2022-04-13

**Authors:** Kyle Fiorini, Tanya Tamasi, Justin Dorie, Ahmed F. Hegazy, Ting-Yim Lee, Marat Slessarev

**Affiliations:** ^1^Schulich School of Medicine and Dentistry, Western University, London, ON, Canada; ^2^Division of Critical Care, Department of Medicine, London Health Sciences Centre, London, ON, Canada; ^3^Kidney Clinical Research Unit, Lawson Health Research Institute, London, ON, Canada; ^4^Department of Anesthesia and Perioperative Medicine, London Health Sciences Centre, London, ON, Canada; ^5^Department of Medical Imaging and Biophysics, Western University, London, ON, Canada; ^6^Imaging Program, Lawson Health Research Institute, London, ON, Canada; ^7^Brain and Mind Institute, Western University, London, ON, Canada

**Keywords:** critical care, heart arrest, hypothermia, induced, brain injuries, thermometers, technology

## Abstract

**Importance:**

Accurate monitoring of core body temperature is integral to targeted temperature management (TTM) following cardiac arrest. However, there are no reliable non-invasive methods for monitoring temperature during TTM.

**Objectives:**

We compared the accuracy and precision of a novel non-invasive Zero-Heat-Flux Thermometer (SpotOn™) to a standard invasive esophageal probe in a cohort of patients undergoing TTM post-cardiac arrest.

**Design, Setting, and Participants:**

We prospectively enrolled 20 patients undergoing post-cardiac arrest care in the intensive care units at the London Health Sciences Centre in London, Canada. A SpotOn™ probe was applied on each patient's forehead, while an esophageal temperature probe was inserted, and both temperature readings were recorded at 1-min intervals for the duration of TTM.

**Main outcomes and Measures:**

We compared the SpotOn™ and esophageal monitors using the Bland–Altman analysis and the Pearson correlation, with accuracy set as a primary outcome. Secondary outcomes included precision and correlation. Bias exceeding 0.1°C and limits of agreement exceeding 0.5°C were considered clinically important.

**Results:**

Sixteen (80%) of patients had complete data used in the final analysis. The median (interquartile range) duration of recording was 38 (12–56) h. Compared to the esophageal probe, SpotOn™ had a bias of 0.06 ± 0.45°C and 95% limits of agreement of −0.83 to 0.95°C. The Pearson correlation coefficient was 0.97 (95% confidence interval 0.9663–0.9678), with a two-tailed *p* < 0.0001.

**Conclusion and Relevance:**

The SpotOn™ is an accurate method that may enable non-invasive monitoring of core body temperature during TTM, although its precision is slightly worse than the predefined 0.5°C when compared to invasive esophageal probe.

## Introduction

Implementation of targeted temperature management (TTM) protocols is an important component of post-cardiac arrest care that is associated with improved neurologic outcomes (1, 2). TTM protocols generally include rapid cooling, maintenance, rewarming, and hyperthermia prevention stages that are delivered over 24–72 h following cardiac arrest (3–5). Recent evidence suggests that targeted normothermia and prevention of hyperthermia in a subset of patients within the post-cardiac arrest period may be as effective as hypothermia in improving post-cardiac arrest outcomes (6). Accurate monitoring of core body temperature is a critical component of TTM, and current guidelines recommend using invasive esophageal, nasopharyngeal, bladder, endotracheal cuff, or pulmonary artery temperature sensors to achieve this goal (3–5).

A novel temperature monitor (SpotOn™, 3M, Minnesota, USA) is an attractive non-invasive alternative for tracking core body temperature during TTM. SpotOn™ is applied to the forehead, creates an isothermic tunnel under the measurement site by insulating heat loss from the skin surface, and estimates core body temperature using the zero-heat-flux technology (7). In perioperative settings, SpotOn™ showed good correlation, accuracy, and precision when compared to esophageal, nasopharyngeal, and pulmonary artery sensors (8–10) and was better than bladder temperature sensors (10). However, these studies only measured temperatures for up to 9 h and did not compare the SpotOn™ monitor during TTM, which employs wider temperature ranges and longer measurement duration (up to 72 h).

The aim of this study was to assess the accuracy, precision, and correlation of the SpotOn™ monitor compared to a standard invasive core body temperature monitor in cardiac arrest patients undergoing TTM.

## Materials and Methods

### Study Design and Patients

This was a prospective observational single-center study that recruited consecutive patients with cardiac arrest admitted to two intensive care units (ICUs) at the London Health Sciences Centre in London, Canada. Inclusion criteria were age ≥18 years with in- and out-of-hospital cardiac arrest who were eligible for TTM as per established hospital protocols developed in accordance with the current practice guidelines (3). Exclusion criteria were immediate plan to withdraw life-sustaining measures, inability to obtain consent, skin rash or infection over the forehead, or medical tape allergy. The study was approved by the Western University Health Sciences Research Ethics Board (Protocol # 109432). We obtained signed informed consent from substitute decision-makers prior to commencing study procedures.

### Study Procedures

Following study enrollment, we initiated simultaneous recording of the patient's core body temperatures using SpotOn™ and esophageal temperature probes for the duration of the TTM protocol. The TTM protocol at our hospital included cooling, maintenance, rewarming, and hyperthermia prevention for up to 72 h following cardiac arrest. In each patient, we used the pressure-sensitive adhesive to secure the SpotOn™ temperature probe to the patient's forehead as per the product monograph. We then connected the probe to the SpotOn™ central portable console and initiated temperature measurement. All patients had esophageal temperature probes inserted as per our institutional TTM protocol. We used an automated data capture module (MediCollector^®^, USA) to simultaneously record temperatures from both the SpotOn™ and esophageal temperature probes at 1-min intervals for the duration of the TTM protocol. We used case report forms for each patient to record demographic data, type of cardiac arrest, comorbidities, duration of TTM protocol, and temperature recording from the medical chart.

### Data Analysis and Statistics

Statistical analysis was performed using GraphPad Prism version 8.3 (GraphPad Software^®^, San Diego, CA, USA). Continuous variables were reported using medians and interquartile ranges (IQRs) for non-normally distributed data or means and standard deviations for normally distributed data, and categorical variables were reported as frequencies (%). The primary endpoint for this study was accuracy between the SpotOn™ and esophageal temperature probes, measured as bias using the Bland–Altman analysis. Secondary outcomes include precision, measured as limits of agreement using the Bland–Altman analysis, and correlation, measured using the Pearson correlation coefficient.

## Results

**Figure 1:**
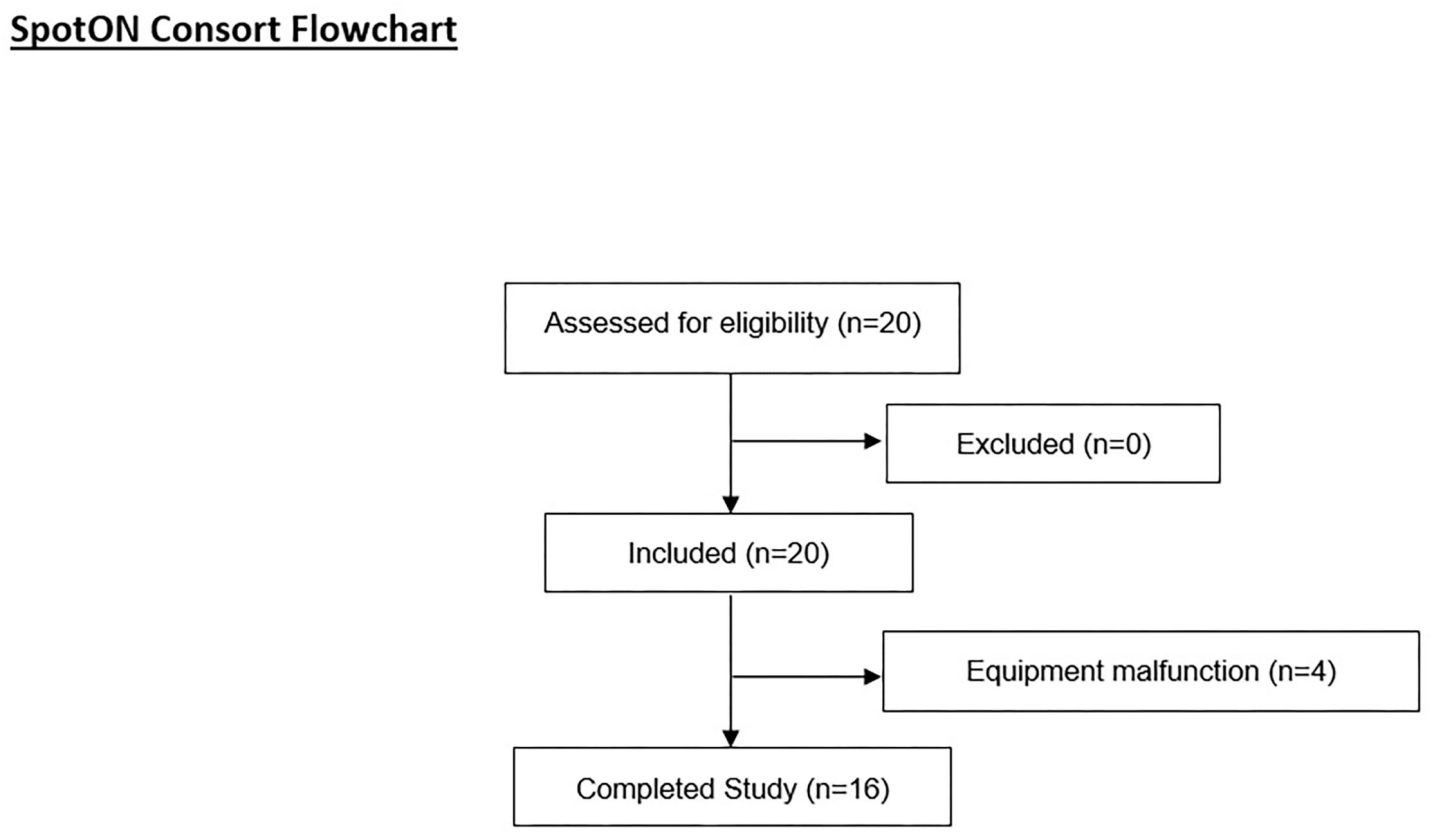
Enrolment flow diagram.

**Figure 2:**
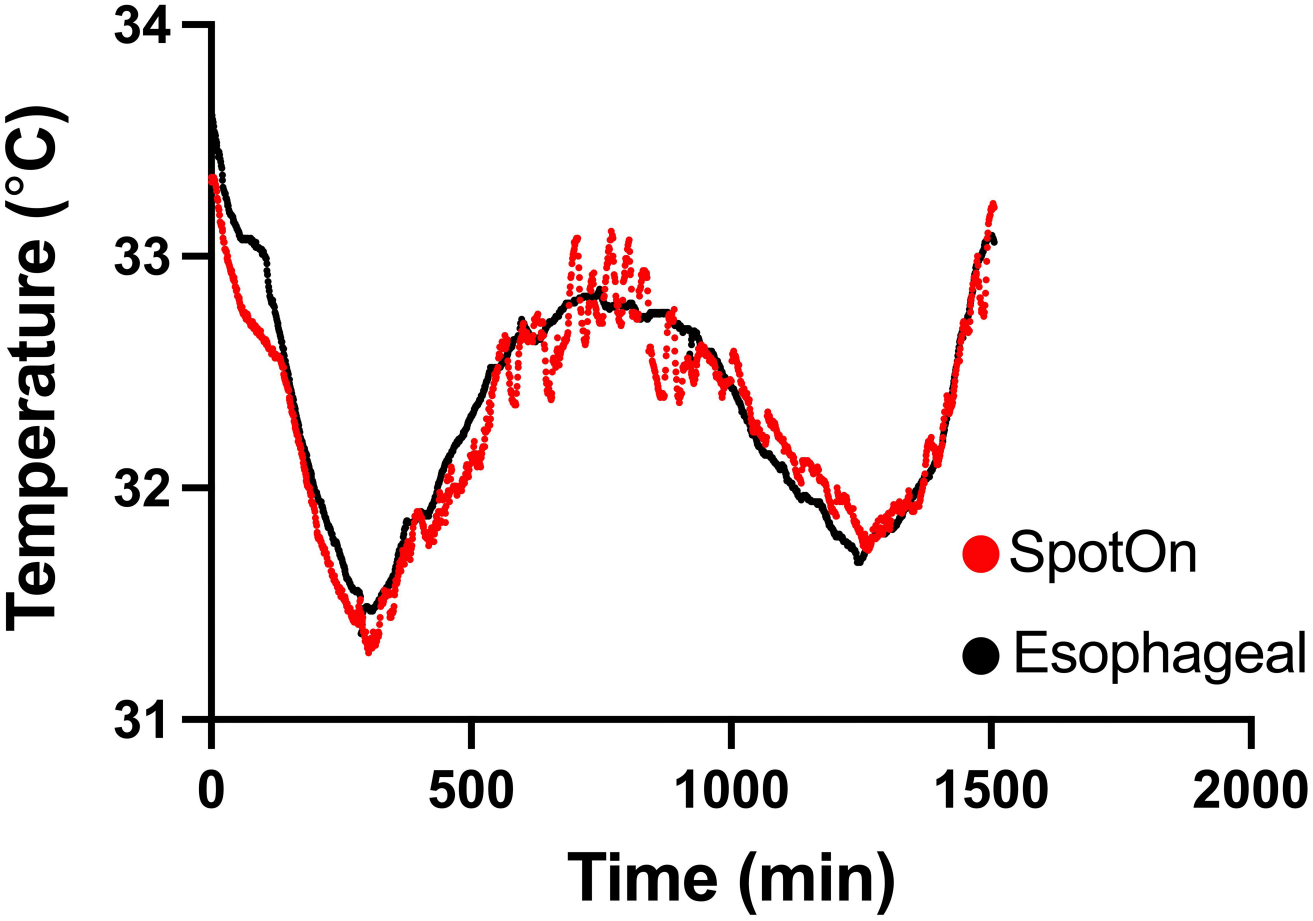
Temperature measurement between SpotOn™ and an esophageal probe in a representative patient.

We enrolled 20 consecutive patients in the study. Four (20%) patients had incomplete data due to failure of the recording equipment and were excluded from data analysis. The remaining 16 patients (5 women) were included in the final analysis (Figure 1). Their clinical characteristics are summarized in Table 1. Median (IQR) age was 66 years (54–72) years, 9 (56%) patients had an out-of-hospital cardiac arrest, 4 (25%) had a shockable rhythm, 8 (50%) had pulseless electrical activity, and the three most common comorbidities were hypertension (56%), dyslipidemia (38%), and diabetes (31%). The median (IQR) duration of the TTM protocol was 24 (24–24) h and the median recording time was 38 (12–56) h. Figure 2 shows a sample recording from a representative patient.

**Table 1:** Patient demographics.

**Subjects**	**16**
Sex (M:F)	11:5
Age (IQR)	65.5 (54–71.5)
IHCA	7 (43.8%)
OHCA	9 (56.2%)
VT or VF	4 (25%)
PEA or asystole	12 (75%)
Cooling—hours (IQR)	24 (24–24)
Recording—hours (IQR)	38.14 (11.69–56.10)
**Comorbidities**	
Coronary disease	0 (0%)
Congestive heart failure	3 (18.75%)
Diabetes	5 (31.25%)
Hypertension	9 (56.25%)
Chronic kidney disease	3 (18.75%)
Dyslipidemia	6 (37.5%)
COPD	2 (12.5%)

*IQR, interquartile range; IHCA, in-hospital cardiac arrest; OHCA, out-of-hospital cardiac arrest; VT, ventricular tachycardia; VF, ventricular fibrillation; PEA, pulseless electrical activity; COPD, chronic obstructive pulmonary disease*.

**Figure 3:**
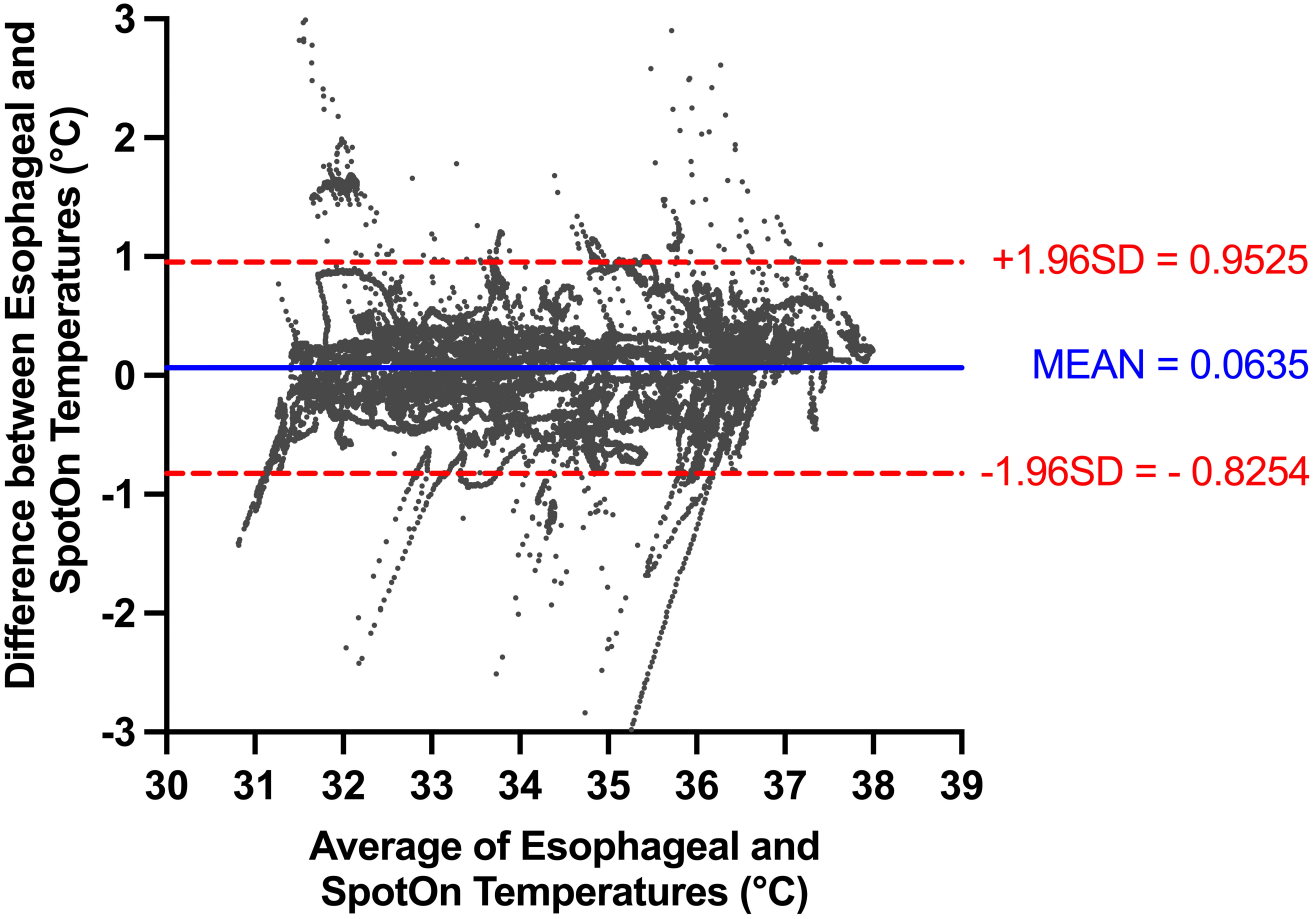
Bland-Altman analysis comparing SpotOn™ and the esophageal temperature probe.

**Figure 4:**
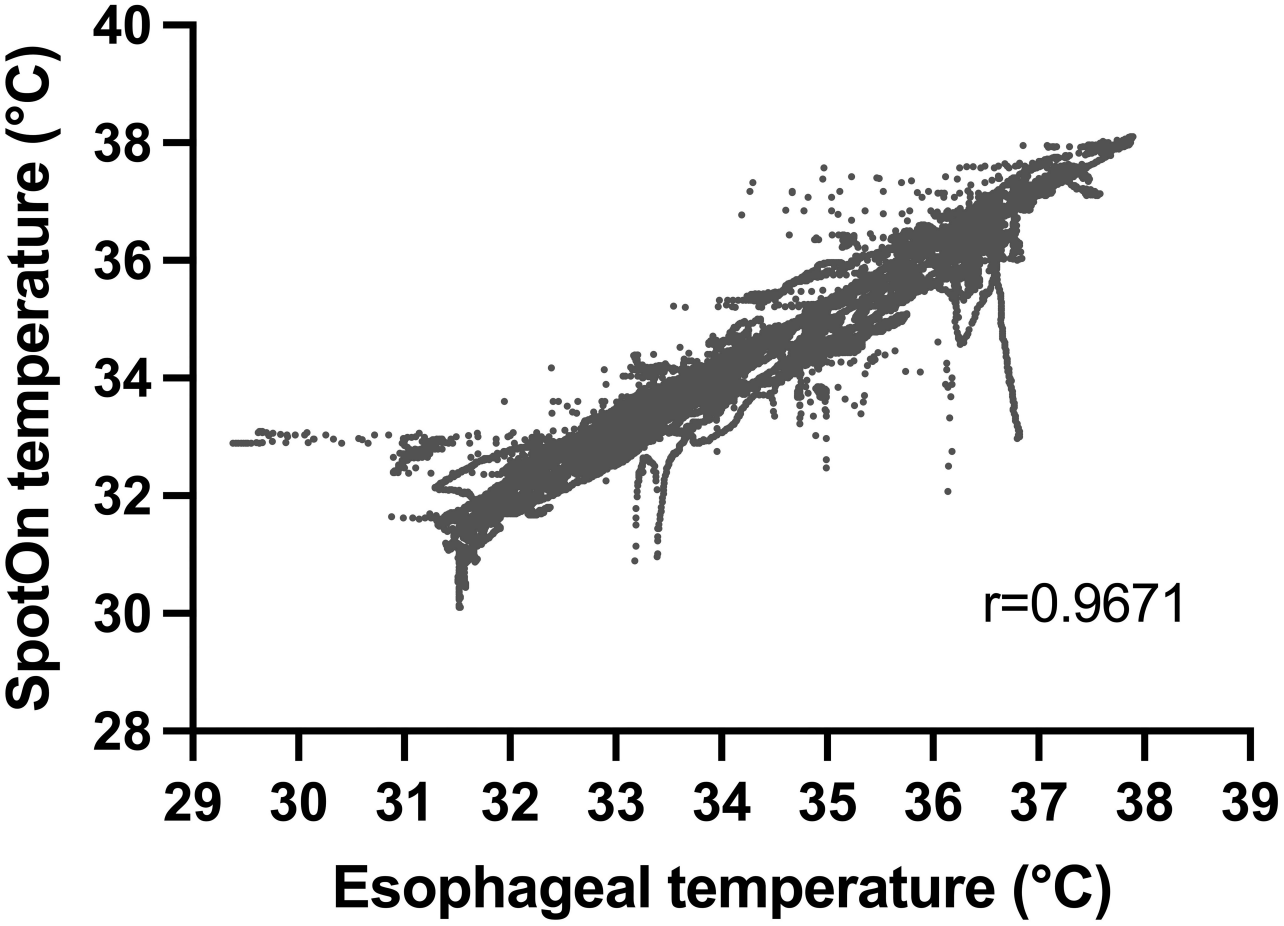
Correlation between temperature measurement using SpotOn™ and the esophageal temperature probe.

Combining data across all patients with multiple observations per patient, we had 29,750 sets of data points from the two temperature probes. All patients within the study underwent rapid cooling as part of TTM, although their temperature target varied at the discretion of the treating physician. Temperatures detected by the esophageal probe ranged from 29.4 to 37.9°C. Within this range, there were no temperatures that were not able to be detected by the SponOn™ probe. For our primary outcome (accuracy), the bias measured using the Bland–Altman analysis was 0.06 ± 0.45°C (Figure 3). For our secondary outcomes, the precision as measured by the Bland–Altman analysis 95% limits of agreement was −0.83 to 0.95°C (Figure 3). The correlation analysis between the two modalities showed a Pearson coefficient of 0.967 (95% confidence interval 0.966–0.968), with a two-tailed *p* < 0.0001 (Figure 4).

**Figure 5:**
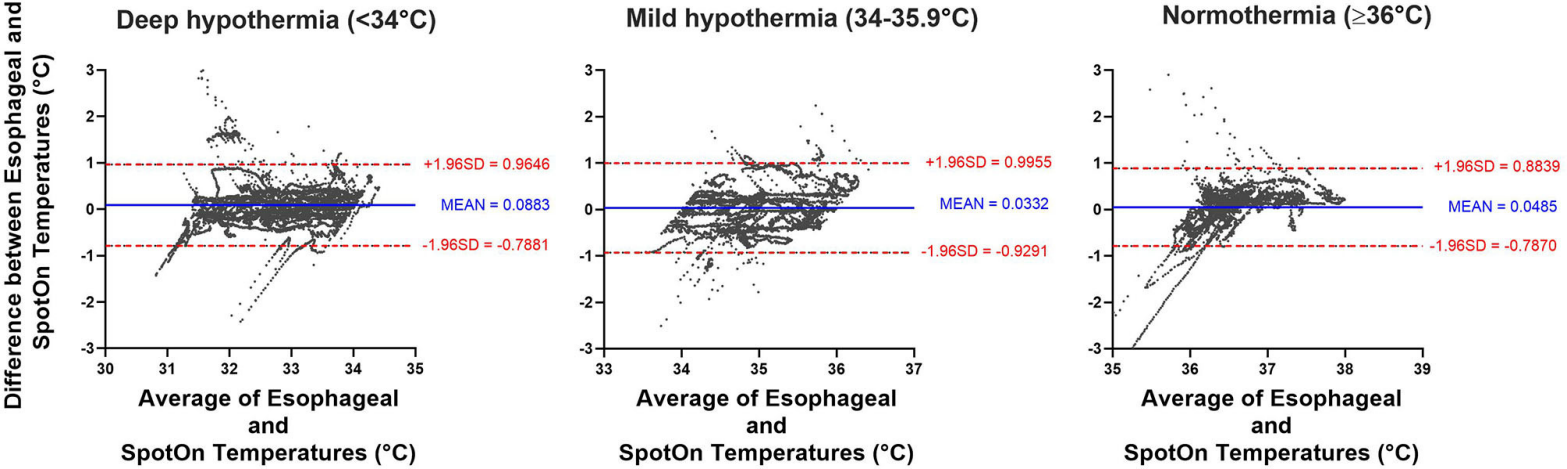
Bland-Altman analysis comparing SpotOn™ and esophageal temperature probes in different temperature ranges, including deep hypothermia (<34°C), mild hypothermia (34–35.9°C), and normothermia (≥36°C).

Furthermore, we stratified the data to determine the accuracy and precision of the SpotOn™ temperature probe across three temperature ranges: normothermia (≥36.0°C), mild hypothermia (34.0–35.9°C), and deep hypothermia (<34.0°C). In the normothermia group (≥36.0°C), there were 8,226 pairs of temperature measurements, with a bias of 0.05 ± 0.43°C and 95% limits of agreement of −0.79 to 0.88°C. In the mild hypothermia group (34.0–35.9°C), there were 7,417 pairs of temperature measurements with a bias of 0.03 ± 0.49°C and 95% limits of agreement of 0.93–1.00°C. In the deep hypothermia group (<34.0°C), there were 14,107 pairs of temperature measurements with a bias of 0.09 ± 0.45°C and 95% limits of agreement of −0.79 to 0.97°C (Figure 5).

## Discussion

Targeted temperature management is a fundamental component of post-cardiac arrest care that relies on accurate and precise monitoring of core body temperature (3–5). Current guidelines recommend using esophageal, nasopharyngeal, bladder, endotracheal cuff, and pulmonary artery temperature sensors for monitoring of core body temperature during TTM (3–5). However, these monitors are invasive and may not be readily available in all centers. In this study, we measured the accuracy and precision of the SpotOn™ against the invasive esophageal probe in patients undergoing TTM. Our results suggest that the SpotOn™ monitoring device has an accuracy consistent with what was acceptable in prior studies (11), although its precision is just outside the previously accepted 95% limits of agreement of <0.5°C (9).

The SpotOn™ thermometer has acceptable accuracy and precision for continuous core body temperature monitoring in perioperative settings (8–10) and during TTM (12, 13). Our work compliments these studies by providing a larger dataset spanning broader temperature ranges during TTM (13). Although they have become standard of care, invasive temperature monitors still have associated risks. For example, the risks associated with esophageal probes include vomiting and aspiration, tracheal misplacement and endotracheal tube cuff leak, and mucosal injury that can lead to bleeding, especially in patients with post-cardiac arrest due to concurrent acidosis, hypothermia, and use of therapeutic anticoagulation and platelet inhibitors (14). In contrast, the SpotOn™ monitor is non-invasive and easy to apply. The only potential drawbacks of the SpotOn™ device include restrictions with application in patients with adhesive allergies and potential for skin breakdown with extensive use, although these adverse events were seen neither in our study nor in a prior study that used SpotOn™ for the duration of time that was similar to TTM protocols (13).

An added benefit of the SpotOn™ thermometer is that its placement over the forehead provides an indirect measure of the brain temperature. Given that the purpose of TTM is to limit secondary brain injury by accurately titrating brain temperature, non-invasive monitoring of brain temperature by the SpotOn™ thermometer may be more clinically relevant than monitoring of core body temperature at non-brain sites. In the future, selective brain cooling while maintaining body normothermia may enable neurologic benefits of TTM while minimizing systemic side effects associated with whole-body cooling (15). In these cases, non-invasive monitoring of brain temperature using SpotOn™ would be highly relevant.

Our study had several limitations. We enrolled only 20 patients at a single-center and 4 patients were excluded due to a failure of data recording equipment. While the remaining 16 patients is a small sample size, long duration and high frequency of temperature recording resulted in a large dataset spanning a broad range of temperatures, enabling a robust comparison between the two temperature monitoring modalities. Although this large dataset of paired measurements derives a significant statistical power in determining the agreement between the two devices, one limitation is that our patient sample size was too small to stratify our results into clinical subgroups. Future larger studies can assess the accuracy and precision of the SpotOn™ temperature probe during TTM among clinical subgroups stratified by age, sex, and body mass index.

While the “gold standard” for temperature monitoring is considered the pulmonary artery catheter (16), we compared SpotOn™ performance against the esophageal temperature probe since pulmonary artery catheters are no longer used routinely in patients with post-cardiac arrest in our units. The precision and accuracy of the esophageal temperature probe relative to the pulmonary artery catheter have been previously demonstrated in another study (16). The esophageal temperature probes were placed using standard operating procedures for our ICU and were confirmed using chest X-ray. While one study showed that incorrect position of nasopharyngeal probe placement can affect accuracy of temperature measurement by approximately 0.2°C (17), confirming accurate placement of our esophageal probes beyond chest X-ray using modalities, such as CT scanning, was beyond the standard of care in our patients with post-cardiac arrest. Considering these limitations, we chose to use esophageal probes as a reference standard instead of pulmonary artery catheters.

Another possible limitation of our study was a small proportion of outlying data included in our analysis. The main cause of this was due to dislodgement or detachment of either the SpotOn™ or the esophageal temperature probe. Unfortunately, this occurs commonly during routine post-cardiac arrest care due to nursing care, imaging studies, invasive procedures, and interventions. Given that we were measuring temperatures every minute, even a short period of probe dislodgement or detachment can lead to a sizable number of outliers. However, given that we had an extensive number of data points, the amount of outlying data was not likely significant enough to have a substantial impact on the results. Lastly, shivering may also impact the accuracy of SpotOn™ measurements, although we did not assess this specifically in our study.

## Conclusion

The SpotOn™ monitor is an accurate method for continuous temperature monitoring in patients with post-cardiac arrest, although its precision is slightly worse than the predefined 0.5°C when compared to the invasive esophageal probe. This device is an encouraging alternative to invasive probes for monitoring core body temperature during targeted temperature management, but larger studies will be required to confirm this in various patient populations. Furthermore, the possibility of indirect brain temperature measurement warrants further investigation.

## Data Availability Statement

The raw data supporting the conclusions of this article will be made available by the authors, without undue reservation.

## Ethics Statement

The studies involving human participants were reviewed and approved by Western University Health Sciences Research Ethics Board. The patients/participants provided their written informed consent to participate in this study.

## Author Contributions

TT, JD, and MS were responsible for data collection. KF, MS, and AH performed the data analysis. KF and MS wrote the first draft of the manuscript. All authors contributed to the review and revisions of the manuscript. All authors contributed to the article and approved the submitted version.

## Conflict of Interest

The authors declare that the research was conducted in the absence of any commercial or financial relationships that could be construed as a potential conflict of interest.

## Publisher's Note

All claims expressed in this article are solely those of the authors and do not necessarily represent those of their affiliated organizations, or those of the publisher, the editors and the reviewers. Any product that may be evaluated in this article, or claim that may be made by its manufacturer, is not guaranteed or endorsed by the publisher.
